# Using the transit of Venus to probe the upper planetary atmosphere

**DOI:** 10.1038/ncomms8563

**Published:** 2015-06-23

**Authors:** Fabio Reale, Angelo F. Gambino, Giuseppina Micela, Antonio Maggio, Thomas Widemann, Giuseppe Piccioni

**Affiliations:** 1 Dipartimento di Fisica e Chimica, Università di Palermo, Piazza del Parlamento 1, Palermo 90134, Italy; 2 INAF/Osservatorio Astronomico di Palermo, Piazza del Parlamento 1, Palermo 90134, Italy; 3 Universitè de Versailles-Saint-Quentin, ESR/DYPAC EA 2449, Observatoire de Paris, LESIA, UMR CNRS 8109, 5 Place Jules-Janssen, Meudon 92190, France; 4 INAF-IAPS (Istituto di Astrofisica e Planetologia Spaziali), via del Fosso del Cavaliere 100, Roma 00133, Italy

## Abstract

During a planetary transit, atoms with high atomic number absorb short-wavelength radiation in the upper atmosphere, and the planet should appear larger during a primary transit observed in high-energy bands than in the optical band. Here we measure the radius of Venus with subpixel accuracy during the transit in 2012 observed in the optical, ultraviolet and soft X-rays with Hinode and Solar Dynamics Observatory missions. We find that, while Venus's optical radius is about 80 km larger than the solid body radius (the top of clouds and haze), the radius increases further by >70 km in the extreme ultraviolet and soft X-rays. This measures the altitude of the densest ion layers of Venus's ionosphere (CO_2_ and CO), useful for planning missions *in situ*, and a benchmark case for detecting transits of exoplanets in high-energy bands with future missions, such as the ESA Athena.

## Abstract (web summary)

The atmosphere of a transiting planet shields the stellar radiation enabling size and density stratification to be estimated. Here, the authors study Venus and show that the measured radius depends on the wavelength used, which has implications for Venus's ionosphere and may help in planning future missions.

## Graphical abstract

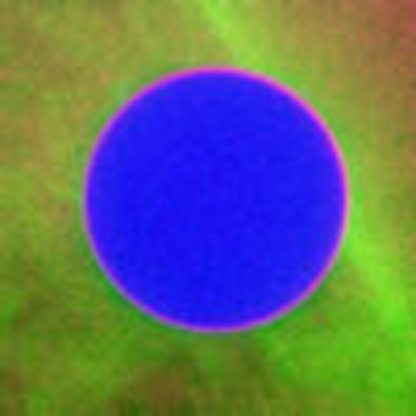

Transits of Venus^1^ are among the rarest of predictable astronomical phenomena. They were once of great scientific importance as they were used to gain the first realistic estimates of the size of the Solar System in the eighteenth to nineteenth centuries^2^. The transit of Venus in 2012 was the last one of the twenty-first century. Here we describe how observations of this last transit made by space missions at ultraviolet, extreme ultraviolet and X-ray wavelengths give us new insight into the upper atmosphere of the planet.

The X-rays and extreme ultraviolet solar radiation is stopped in the ionosphere of rocky planets around the peak of the ion/electron density. Detailed models of the ionosphere structure with the altitude and the solar zenith angle have been developed for Venus^3^,^4^,^5^,^6^,^7^,^8^, and a radio measurement of the electronic density has been obtained^9^. The photoionization induced by the solar extreme ultraviolet radiation produces 
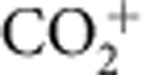
 ions that are transformed by photochemistry to 
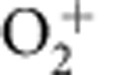
 by reaction with the O^6^,^10^. Models predict that the peak density of 
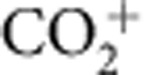
 and other molecular ions should be around 150–180 km^8^. The mass spectrometer on board the Pioneer Venus Orbiter measured the ion composition *in situ* down to periapsis near 150 km, including 
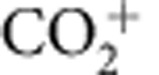
11. Despite at the limit of the observation, the peak at low solar zenith angles appeared to be slightly below 150 km and it should be reasonably similar near the terminator.

The knowledge of the density in the ionosphere and neutral atmosphere is very important for planning the minimum altitude of the fly-by with spacecrafts and entry probes of Venus, since they are subject to dynamical pressure and also electrical charging from the environment. For example, on June 2014, ESA planned for the first time an aerobraking with the Venus Express spacecraft that flew down to a minimum altitude of 129.2 km over the mean surface of Venus yielding a maximum dynamic pressure of more than 0.75 N m^−2^ (ref. 12).

Here we measure the radius of Venus during the transit of 2012 in observations at progressively shorter wavelength from the optical to the X-rays. While the optical radius of ∼6,130 km is in agreement with previous measurements, the values retrieved in the extreme ultraviolet (∼100–300 Å) and X-ray bands (∼10 Å) are significantly larger than the optical radius by 70–100 km, while the altitude in the ultraviolet band (∼1,500 Å) is in between (40–50 km). Our study of the Venus transit will be useful for planning and interpreting future data: the topic of planetary atmospheres will be certainly pursued very actively with future instrumentation (James Webb Space Telescope (JWST), Athena and so on), because of its relevance for understanding planet physics and life conditions in the Universe. In the mean time, our measurements are providing new information for explaining the drag that the planet atmosphere exerts on space probes currently in orbit around Venus.

## Results

### The observations

**Figure 1:**
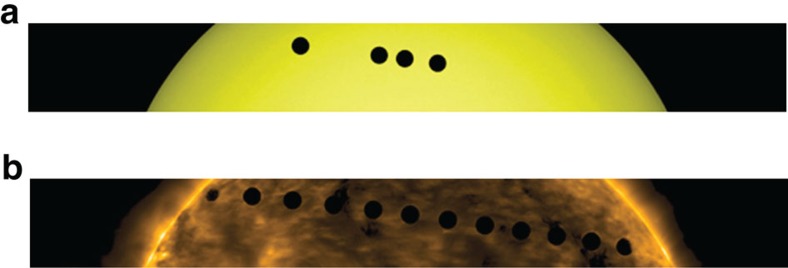
Path of Venus's transit across the solar disk. Path (**a**) in the optical (4,500 Å) band and (**b**) in the extreme ultraviolet (171 Å) band of the Atmospheric Imaging Assembly on board the Solar Dynamics Observatory. The planet disks span the range of the selected data.

The transit of Venus started on 5 June 2012 at 22:25 UTC and ended on 6 June 2012 at 04:16 UTC (third contact), during a period of moderate solar activity. It has been observed in great detail by space-borne solar observatories, and in particular by imaging instruments on-board Hinode^13^ and Solar Dynamics Observatory^14^ (Fig. 1). Here we analyse the observations of the Solar Dynamics Observatory/Atmospheric Imaging Assembly (AIA)^15^ in the optical (4,500 Å), ultraviolet (1,600 and 1,700 Å) and extreme ultraviolet (171, 193, 211, 304 and 335 Å) channels, and of the Hinode/X-Ray Telescope (XRT)^16^ in the Ti-poly filter that has the maximum sensitivity at ∼10 Å (Table 1). The AIA and XRT plate scales are 0.60000, arcsec per pixel and 1.0286, arcsec per pixel, respectively.

### Venus's radii

**Figure 2:**
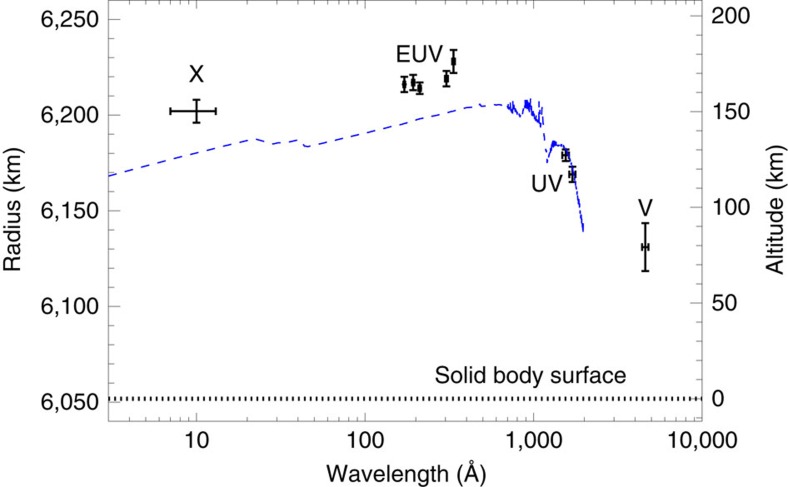
Radius of Venus measured from the transit as a function of the wavelength. Radius values (data points) from the X-rays (X) to the extreme ultraviolet (EUV), ultraviolet and optical (V) band. The approximate full-width-half-maximum of each channel^16^,^24^ is marked (horizontal error bar). Predictions from a model^8^ at SZA=90° (blue dashed line) is shown for comparison.

The values of the radius are shown in Table 1 and in Fig. 2. We see that the radius values follow a well-defined trend. Venus's mean solid body radius is *R*_sf_=6,051.8±1.0 km from the cartographic reference system obtained with Magellan^17^. The optical radius is in agreement with the mean cloud top altitude of 74±1 km retrieved from Venus Express/Visible and InfraRed Thermal Imaging Spectrometer (VIRTIS)^18^, and more specifically, with the expected opacity mainly due to upper haze in the first scale height above cloud tops. VEx/SOIR results^19^ place that altitude at 73±2 km in the 3 μm band, to be further increased by 6±1 km to retrieve its value in the visible domain^19^,^20^. At higher energies we measure larger altitudes, by 40–50 km in the ultraviolet band, up to 70–100 km in the extreme ultraviolet and X-ray bands.

### Atmosphere altitudes

The altitude that we measure in the extreme ultraviolet and X-ray bands corresponds to the height where the optical thickness along the tangential line of sight reaches the value *τ*=1. At this altitude the solar radiation is mostly absorbed by photoionization of neutral atoms, and hence this is also the altitude where a peak of the electron density is expected. In particular, the *F*_1_ peak in density profiles of planetary atmospheres is associated with the absorption of extreme ultraviolet radiation^8^, while ultraviolet photons and soft X-rays are absorbed in slightly deeper layers. We have verified that the different Venus sizes indicated by ultraviolet and extreme ultraviolet data can be explained in terms of tangential column densities at the retrieved altitudes (165–170 km for extreme ultraviolet and 125–135 km for ultraviolet from the solid body) at the terminator between the day and night hemispheres (solar zenith angle (SZA), 90°). The absorption path length, *l*, at a given altitude *h* can be expressed as 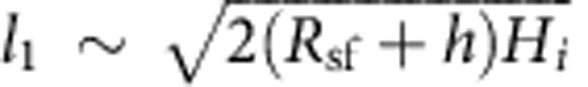, where *H*_*i*_ is the atmospheric scale height of each molecular species *i*, and the optical thickness can be evaluated as *τ*(*λ*)=∑*n*_*i*_*σ*_*i*_(*λ*)*l*_*i*_, where *n*_*i*_ and *σ*_*i*_(*λ*) are the molecular density and absorption cross section, respectively. At 170 km CO_2_ and CO have approximately the same mixing ratio^21^, while at 130 km CO_2_ dominates. A simple calculation assuming a spherical geometry shows that the neutral atmosphere is ∼20 times more opaque in the extreme ultraviolet than in the ultraviolet at 170 km and ∼80 times at 130 km in the regions probed.

Figure 2 shows a comparison between our ionospheric altitudes measured at different wavelengths with the prediction from a detailed model that includes also a dependence on the SZA^8^. There is a very good general agreement and an accurate one at ultraviolet wavelength. However, we find that at extreme ultraviolet and X-ray wavelengths, although consistent with soft X-rays absorbed at slightly deeper layers than extreme ultraviolet^8^, Venus's atmosphere appears more opaque than expected from the model for SZA=90°. This result indicates a tangential column density at heights *h*∼150–170 km larger than expected from the model in spherical geometry at the terminator, that is, a higher neutral density than predicted by the standard model of Venus's atmosphere^21^ or a longer path. The latter might be the case if the ionosphere at large heights deviates from spherical geometry due to the pressure of the solar wind. This does not seem to be the case deeper in the atmosphere where the ultraviolet radiation is absorbed.

## Discussion

This is the first time that such a multi-wavelength measurement has ever been performed for a Solar System body, and it is a very rare, unplanned opportunity we caught; in fact, the occurrence of a transit of a planet with an atmosphere over the Sun and the simultaneous availability of optical, ultraviolet and X-ray observations will not happen again in the near future. This measurement is useful as a check for models of Venus's atmosphere^7^,^9^,^10^ at the terminator, where large changes are occurring due to the transition from sunlight to darkness^8^. It also provides an independent measurement of the molecular column at heights that were recently analysed by tracking data of the Venus Express Atmospheric Drag Experiment^22^ in complement to the neutral mass spectrometer instrument on-board Pioneer Venus Orbiter (PVO) and Magellan orbital drag measurements. On the other hand, multi-wavelength (infrared, optical and X-ray) observations of transits of planets in extra-solar systems are already within the possibilities of current observing facilities on Earth and from space, but this methodology is still at its very early phases of assessment for the study of exoplanetary atmospheres.

## Methods

### Summary

We measured the planet radius using the radial intensity profiles from the planet disk centre in each selected image. To improve on accuracy and robustness, we derived the intensity values as latitudinal averages, on annuli centred on the planet disk centre. This allows us to achieve subpixel sensitivity: the thickness of the concentric annuli is 0.2 pixels (25.1 km at the Venus distance) and 0.25 pixels (53.9 km) for AIA and XRT images, respectively. The intensity profiles we obtained have a smoothed limb due to the convolution of a step function with the instrument Point Spread Function (PSF), and are symmetrical around the middle intensity value, as expected for a symmetrical PSF. We considered the occulting radius for a given wavelength channel as the distance between this point at half intensity and the disk centre. For each channel, we obtained as many values of the radius as the number of selected images.

Owing to high signal-to-noise ratio (S/N) in the optical band, retrieved values are constant within the uncertainty margin of 0.1 pixels, corresponding to 12.6 km at the distance of Venus. In all the other bands we obtained normal distributions of radii and, therefore, the centroid position (mean value) as the best radius value and the s.d. of the mean as its uncertainty.

### Image analysis and radius measurement

**Figure 3:**
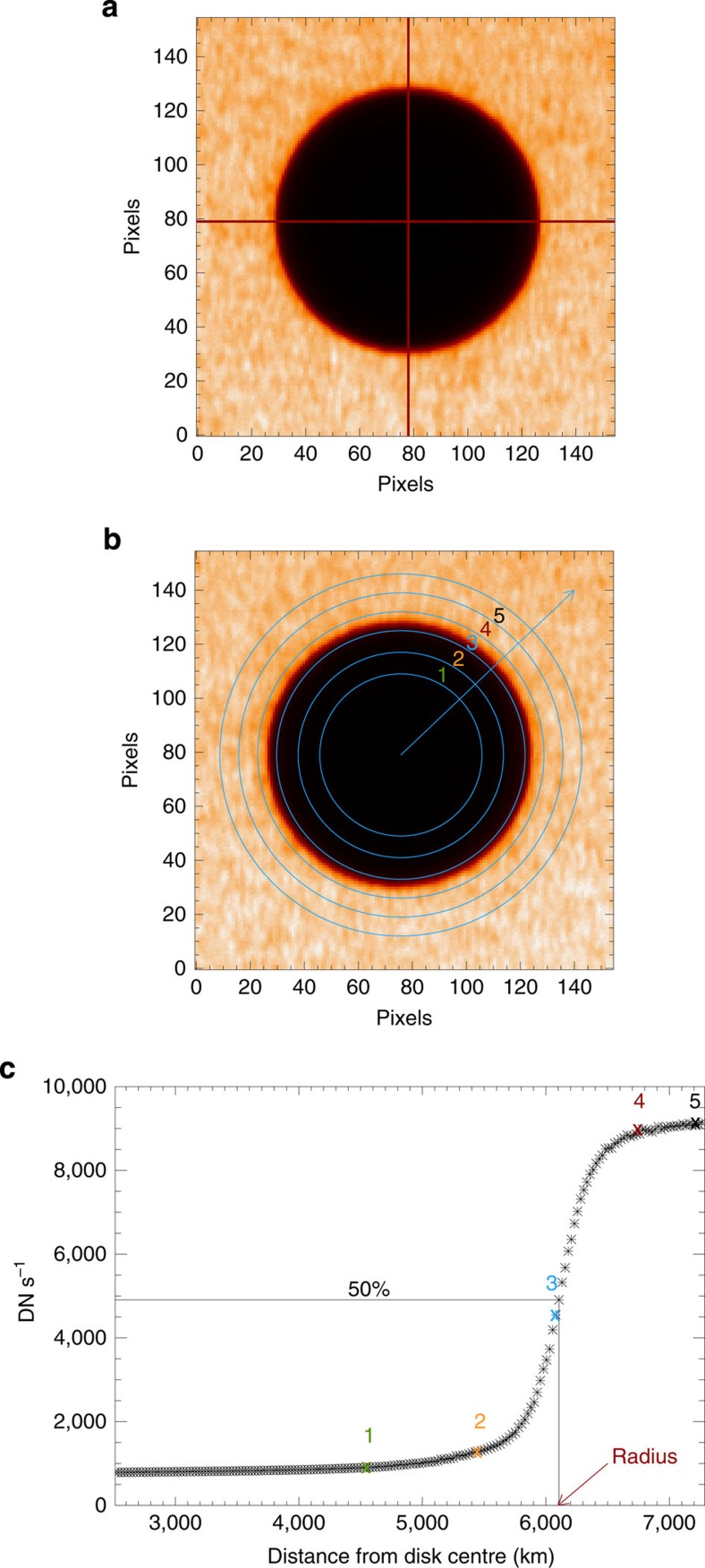
The measurement of the radius. (**a**) Example of the result of the localization of the planet disk centre. (**b**) Planet disk segmented into concentric annuli (not in scale). (**c**) We build the radial intensity profile as the sequence of intensity averages over each annulus. The planet radius is measured as the distance of the data point closest to the 50% intensity level from the disk centre.

For each image in each channel, we measure the radius from the intensity profile as a function of the distance from the planet disk centre. We cut out a square subregion of the image that contains the planet disk. In this subregion, we consider a moving circle with a fixed radius (48 pixels for AIA and 24 for XRT) and we localize the planet disk centre as the one where the average variance of the intensity is the minimum. We obtain a precision of 0.25 pixels along each coordinate. Figure 3a shows an example of the result of this procedure.

Once localized the centre of the planet disk in each image, we have ascertained that the planet disk is a perfect circle within the uncertainties. Then we segment the planet disk into concentric annuli as shown in Fig. 3b. The width of the concentric annuli is 0.2 and 0.25 pixels for AIA and XRT images, respectively, that correspond to 25.1 km and 53.9 km at the Venus distance.

**Figure 4:**
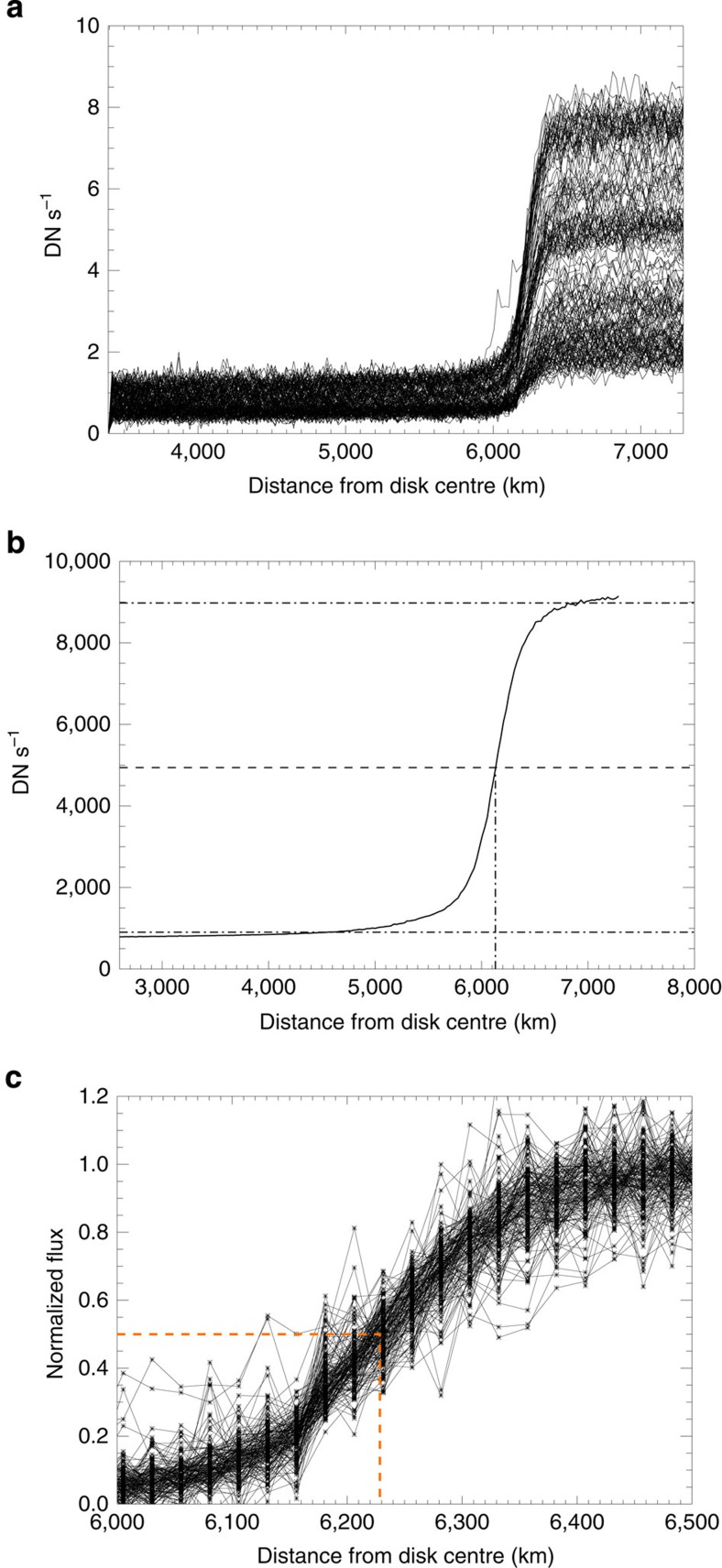
Analysis of the limb profiles. (**a**) Intensity profiles (DN rate per pixel) across the planet disk measured from the planet disk centre in the 335-Å channel (see Fig. 3c). The intensity out of the disk (rightwards) depends on the coronal region the planet is crossing. (**b**) This average intensity profile (4,500 Å) is symmetrical with respect to the middle horizontal line, that is, the 50% level between the out-of-disk intensity and the in-disk intensity (dash-dotted lines). (**c**) Profiles in the 335-Å channel (Fig. 4a) normalized in the range between the high and low values used to evaluate the 50% intensity level. Only the region around the planet limb is shown. The best radius value is marked (orange dashed lines).

For each annulus, we average the intensity of all pixels whose radial distance from the centre is inside the annulus. At the distance of the disk limb, each annulus intercepts ∼50 and ∼40 pixels for AIA and XRT, respectively. We build a profile of the sequence of average intensities as a function of the radial distance from the planet disk centre (Fig. 3c). We obtain an intensity profile for each image. Figure 4 shows all profiles obtained from the observations in the 335-Å channel, overplotted in the same panel. While inside the disk the profiles span a relatively narrow range (0–2 DN s^−1^), the range is much broader outside of the disk (2–8 DN s^−1^), where the intensity depends on the coronal region behind the planet at that moment and the corona is highly inhomogeneous (Fig. 1).

The intensity profiles correspond to a smoothed limb, since they are the result of the convolution of a step function with the instrument PSF, as shown in Fig. 4b. We checked that the profiles are symmetrical around the middle intensity value, as expected for a PSF symmetrical in the core region.

The middle intensity value is found between a high value and a low value taken very far from the planet limb. The high value is the average in a 2-pixel (2.5 pixels for XRT)-wide annulus (that is, we average over ∼1,000 pixels) beyond the outer limb (starting at 6,400–6,500 km from the planet centre). The low value is the average in a broad annulus (∼25 pixels wide, ∼15 for XRT) inside the planet shadow that includes about 7,000 pixels (1,300 pixels for XRT). The large number of pixels involved makes the error on the high and low values negligible. For each profile, we measure the radius as the distance between the data point closest to the middle intensity (that is, 50% between the low- and high-value levels) and the planet centre (Fig. 3c).

### Statistical analysis of the radius values

For each channel, we obtain as many values of the radius as the number of images, listed in Table 2. We obtain distributions of values that depend on the noise level of the signal. In the optical channel, we obtain invariably a value of 48.8±0.1 pixels of the radius, because of a high S/N of ∼10^3^, and the radius variations from one profile to the other are below our measurement margin (Fig. 4b). In this case, we have an upper limit to the uncertainty that is half of our sensitivity, that is, 0.1 pixels that corresponds to 12.6 km at the distance of Venus. In the ultraviolet and extreme ultraviolet channels the S/N is lower, and we obtain statistical distributions of radius values. Figure 4c shows all the profiles in the 335-Å channel (Fig. 4a) normalized to the low and high values used to evaluate the middle 50% level. Only the region around the planet limb is shown. The intensities are sampled at the discrete distances of the annuli from the planet centre, that is, spaced by 25 km. At each annulus distance,the intensity values are scattered (along the *Y*-direction). We have ascertained that this scatter is due to the count statistics. The exposure time is ≈3 s. With an average DN rate of ∼3 DN s^−1^ per pixel (Fig. 4a), we have about ∼400 counts in 50 pixels, with an expected s.d. of ∼20, that is, ∼0.05 in the scale of Fig. 4c. From the average slope of the central part of the profiles in Fig. 4c, this scatter propagates into *σ*_R_∼20 km in the *X*-direction.

**Figure 5:**
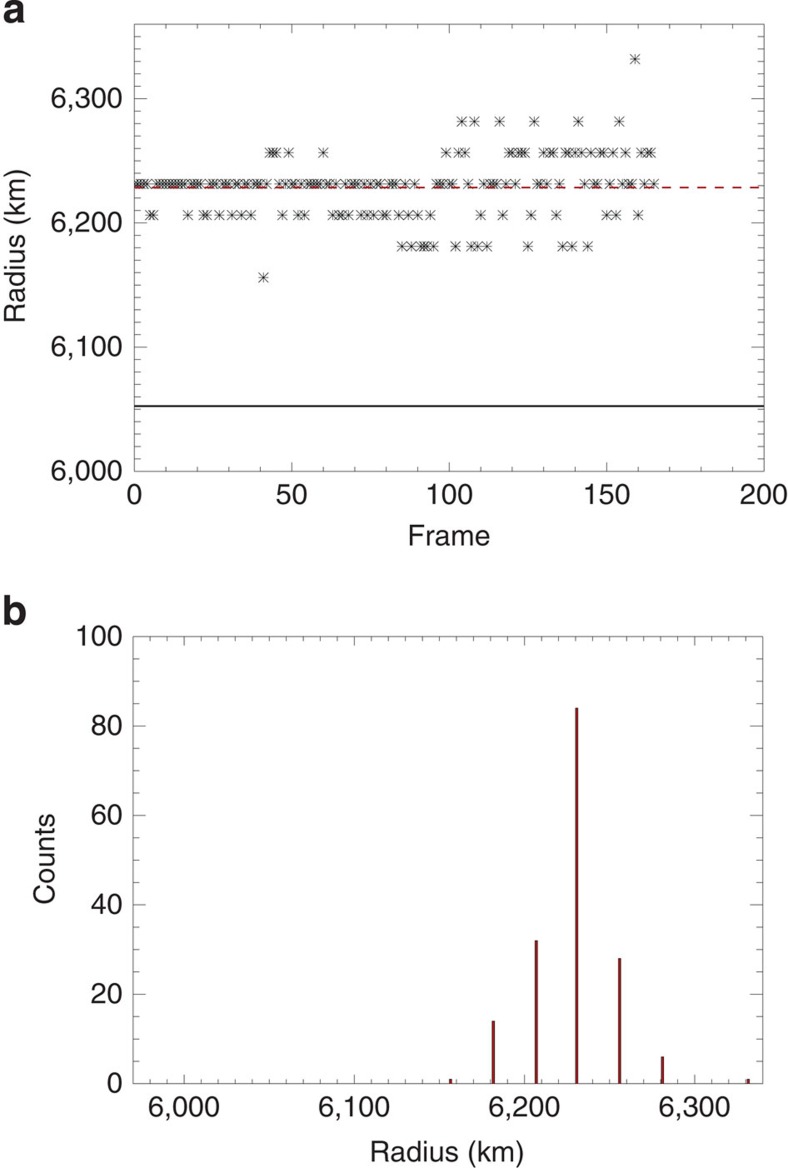
Statistical analysis of the radius values. (**a**) Radius values as a function of the image order number for the extreme ultraviolet 335-Å channel. The dashed and solid lines mark the mean value and the solid body radius, respectively. (**b**) Histogram of the distribution of the radius values in **a**.

Figure 5 shows the distribution of the radii for the 335-Å channel in the form of a scatter plot and of a histogram. The distribution has reasonably the shape of a normal distribution. Table 2 shows the s.d. of the distributions *σ* and the s.d. of the centroid positions *σ*_avg_ in all channels. The value of σ listed in Table 2 for the 335-Å channel is not very different from *σ*_R_ estimated above. The radius values listed in Table 1 are the average values of the radius values obtained from each profile and correspond to the centroid position of the radius distributions. The s.d. of the centroid position *σ*_avg_ is obtained by dividing the distribution width *σ* by the square root of the number of frames listed in Table 2. This corresponds to a confidence level of 68%. The uncertainties on the radius listed in Table 1 are at the 3*σ*=99.73% confidence level. We have ascertained that the possible systematic uncertainty in correcting for the wavelength-dependence of the plate scale (0.000038, arcsec per pixel)^23^ corresponds to a scale of ∼0.4 km and is much smaller than our uncertainty on the radius values listed in Table 1.

## Additional information

**How to cite this article**: Reale, F. *et al.* Using the transit of Venus to probe the upper planetary atmosphere. *Nat. Commun.* 6:7563 doi: 10.1038/ncomms8563 (2015).

## Figures and Tables

**Table 1:** Observations and occulting radii and altitudes of Venus at different wavelengths during the transit of June 2012.

**Band**	***λ*****(Å)**	**Start time UTC (5 June)**	**End time UTC (6 June)**	**Radius (km)**	Altitude versus clouds* **(km)**	**Altitude versus surface (km)**
Optical	4,500	23:30	01:16	6131±13†	0±13†	79±13†
Ultraviolet	1,700	22:26	04:14	6169±4	38±4	117±4
	1,600	22:26	04:14	6179±3	48±3	127±3
Extreme ultraviolet	335	22:23	04:20	6228±6	97±6	176±6
	304	22:23	04:20	6219±4	88±4	167±4
	211	22:23	04:20	6214±3	83±3	162±3
	193	22:23	04:20	6217±4	86±4	165±4
	171	22:23	04:20	6216±4	85±4	164±4
X-rays	10	22:51	04:24	6202±6	71±6	150±6

^*^‘Clouds' is used here as reference altitude for simplicity, since the optical opacity in limb view is actually limited by clouds and haze on top of it without a precise boundary.

^†^Upper limit for the uncertainty estimated as half of our sensitivity, that is, 0.1 pixels, with the SDO/AIA instrument in the optical band.

**Table 2:** Statistical properties of radius distributions at different wavelengths.

**Band**	***λ*****(Å)**	**No. of frames**	σ* **(km)**	***σ***_**avg**_† **(km)**
Optical	4,500	78	‡	‡
Ultraviolet	1,700	114	13.03	1.222
	1,600	124	10.50	0.943
Extreme ultraviolet	335	166	24.96	1.937
	304	118	14.22	1.310
	211	117	12.02	1.111
	193	119	14.86	1.362
	171	120	15.42	1.407
X-rays	10	102	21.07	2.086

^*^s.d.

^†^s.d. of the mean.

^‡^<0.1 pixels.
